# Ecological pleiotropy and indirect effects alter the potential for evolutionary rescue

**DOI:** 10.1111/eva.12745

**Published:** 2018-12-26

**Authors:** John P. DeLong, Jonathan Belmaker

**Affiliations:** ^1^ University of Nebraska – Lincoln Lincoln Nebraska; ^2^ School of Zoology, George S. Wise Faculty of Life Sciences Tel Aviv University Tel Aviv Israel; ^3^ The Steinhardt Museum of Natural History Tel Aviv University Tel Aviv Israel

**Keywords:** allometric population models, eco‐evolutionary dynamics, Gillespie eco‐evolutionary model, invasive predator, paradox of enrichment

## Abstract

Invading predators can negatively affect naïve prey populations due to a lack of evolved defenses. Many species therefore may be at risk of extinction due to overexploitation by exotic predators. Yet the strong selective effect of predation might drive evolution of imperiled prey toward more resistant forms, potentially allowing the prey to persist. We evaluated the potential for evolutionary rescue in an imperiled prey using Gillespie eco‐evolutionary models (GEMs). We focused on a system parameterized for protists where changes in prey body size may influence intrinsic rate of population growth, space clearance rate (initial slope of the functional response), and the energetic benefit to predators. Our results show that the likelihood of rescue depends on (a) whether multiple parameters connected to the same evolving trait (i.e., ecological pleiotropy) combine to magnify selection, (b) whether the evolving trait causes negative indirect effects on the predator population by altering the energy gain per prey, (c) whether heritable trait variation is sufficient to foster rapid evolution, and (d) whether prey abundances are stable enough to avoid very rapid extinction. We also show that when evolution fosters rescue by increasing the prey equilibrium abundance, invasive predator populations also can be rescued, potentially leading to additional negative effects on other species. Thus, ecological pleiotropy, indirect effects, and system dynamics may be important factors influencing the potential for evolutionary rescue for both imperiled prey and invading predators. These results suggest that bolstering trait variation may be key to fostering evolutionary rescue, but also that the myriad direct and indirect effects of trait change could either make rescue outcomes unpredictable or, if they occur, cause rescue to have side effects such as bolstering the populations of invasive species.

## 1 INTRODUCTION

Changes in climate, land use, and species introductions can influence population abundance and may, in some cases, lead to species extinctions (Doherty, Glen, Nimmo, Ritchie, & Dickman, 2016; Jantz et al., 2015; Urban, 2015). This effect may be especially severe for invasive predators, as these predators often are more effective at capturing prey than their native counterparts (Alexander, Dick, Weyl, Robinson, & Richardson, 2014). Without coevolved defensive strategies, naïve prey may go extinct, thereby lowering diversity, altering the structure of native ecological communities, and potentially causing secondary extinctions (Sanders, Kehoe, & van Veen, 2015). It is possible, however, that declining populations will adapt to the changing conditions and persist instead of going extinct, a process known as evolutionary rescue (Bell & Gonzalez, 2009; Carlson, Cunningham, & Westley, 2014; Cotto et al., 2017; Gomulkiewicz & Holt, 1995; Gonzalez, Ronce, Ferriere, & Hochberg, 2013; Jones, 2008; Lindsey, Gallie, Taylor, & Kerr, 2013). Evolutionary rescue is a form of eco‐evolutionary dynamics, wherein evolution alters ecological dynamics, such as those of population size, in ecological time (Pelletier, Garant, & Hendry, 2009; Post & Palkovacs, 2009; Schoener, 2011; Yoshida, Jones, Ellner, Fussmann, & Hairston, Jr., 2003). A typical view of evolutionary rescue is the reversal of a population trajectory deterministically driven toward extinction by a stressor. Alternatively, population extinction through stochastic processes is more likely when populations approach critical levels—a key component of population viability analyses (Lande, 1993; Saunders, Cuthbert, & Zipkin, 2018; Soulé, 1986)—so evolutionary rescue also can be thought of as the situation in which trait evolution reduces the chance of stochastic loss.

Predation imposes strong selective gradients on prey (Losos, Schoener, & Spiller, 2004; Reznick, Bryga, & Endler, 1990; Siepielski, Wang, & Prince, 2014), so it is possible that prey populations could evolve defensive strategies that would limit invading predator effectiveness and rescue their populations before they went extinct (Faillace & Morin, 2016; Gomulkiewicz & Holt, 1995). While robust empirical evidence for evolutionary rescue is still quite rare (Bell & Gonzalez, 2009; Faillace & Morin, 2016; Vander Wal, Garant, Festa‐Bianchet, & Pelletier, 2013), evolutionary rescue of prey in the face of invasive predators appears possible (Phillips & Shine, 2004). For example, following the invasion of an exotic zooplankton predator, native *Daphnia* increased substantially in body size over a 15‐y period (Gillis & Walsh, 2017). This increase in size had a heritable component and was associated with increased population growth in invaded lakes, and therefore, the change in size may have allowed evolutionary rescue of the prey (Gillis & Walsh, 2017). Evolution of imperiled prey also may occur via the reduction in effectiveness of invasive predators. For example, frog tadpoles from populations exposed to an invasive predatory frog were better at avoiding predation compared to populations where the invasive frog was absent (Kiesecker & Blaustein, 1997).

Despite some grounds for optimism about the potential for evolution to rescue imperiled prey and other species exposed to changing environmental conditions, there may be limitations on the effectiveness of this process. Specifically, evolutionary rescue may be influenced by the level of standing heritable variation, which may be insufficient to create fitness differences among individuals in the population (Bell, 2013; Imura, Toquenaga, & Fujii, 2003). In addition, the pace of evolution may lag behind changes in population abundance (DeLong et al., 2016), limiting the potential for rescue. Evolutionary rescue is thought to be most likely when populations are initially large, have high standing genetic variation, and when environmental change is gradual (Carlson et al., 2014; Imura et al., 2003; Vander Wal et al., 2013), but species imperiled by exotic predators are likely to have been driven to low population abundance after a relatively sudden increase in predation risk, limiting the potential for selection due to heightened genetic drift or demographic and individual stochasticity (Bell, 2008; van Daalen & Caswell, 2017). Although in theory, coevolution also can foster rescue of imperiled prey (Jones, 2008; Northfield & Ives, 2013), the potential for evolution in invasive predators may be relatively low compared to the prey. For example, when an invasive predator arrives with only a few individuals, and thus limited genetic variation, predator evolution may lag behind that of their prey. However, some invasive predators, including some that have been intentionally introduced with multiple individuals, have evolved postintroduction, potentially exacerbating their effects on native prey (Phillips, Brown, Webb, & Shine, 2006). Furthermore, the opportunity for evolutionary rescue may depend on the trophic level, species interactions, or temperature, generating considerable uncertainty about whether evolutionary rescue could occur in any given scenario (Kovach‐Orr & Fussmann, 2013; Osmond & de Mazancourt, 2013; Tseng & O'Connor, 2015).

Traits may differ in the way they influence evolutionary rescue. For example, genetic variation in birth rates, mortality, and reproduction can influence evolutionary rescue in different ways (Martin, Aguilée, Ramsayer, Kaltz, & Ronce, 2013), and it is not necessarily the case that changes in the traits under selection will create the right feedback to stabilize the prey population. Traits may be linked to many different functional aspects (captured by model parameters) of predator–prey dynamics or to multiple parameters (i.e., ecological pleiotropy; Strauss & Irwin, 2004; DeLong & Gibert, 2016; DeLong, 2017), leading to net outcomes that might augment or decrease the potential for rescue. Thus, it remains unresolved just whether evolutionary rescue is possible for prey imperiled by exotic predators, what functional processes (i.e., trait‐parameter links) might facilitate it, and how much heritable trait variation is required for a sufficient response to selection to occur before extinction arrives.

Here, we ask (a) whether multiple connections between traits and parameters influence the magnitude of selection and the potential for subsequent rescue, (b) whether indirect effects of prey evolution on predator populations influence the potential for rescue, (c) what levels of heritable trait variation are sufficient to foster rescue, and (d) whether variation in system stability (amplitude of population cycles) influences evolutionary rescue. We employ a new type of eco‐evolutionary model—Gillespie eco‐evolutionary models (GEMs)—to address these questions (DeLong & Gibert, 2016), allowing us to incorporate the critical effects of stochasticity that can lead to both extinction and influence the rate of evolution (Abbott & Nolting, 2017; van Daalen & Caswell, 2017; Lande, 1993). We consider a two‐species consumer–resource scenario in which predators drive prey abundance low enough to cause frequent stochastic extinctions. We fully parameterize the model and the trait‐parameter linkages using an extensive database on consumer–resource interactions for protists.

## 2 METHODS

### 2.1 Model

We envisioned a consumer–resource scenario in which an invasive predator can induce stochastic extinction of its prey after suppressing its population below some previously high level. We used a standard consumer–resource model that represents prey births with the logistic growth equation, has a type II functional response with mutual interference, and a linear predator death term (Hassell & Varley, 1969; Rosenzweig & MacArthur, 1963):

(1A) $$\frac{\text{dR}}{\text{dt}}=rR\left(1-\frac{R}{K}\right)-\frac{aRC^{m+1}}{1+ahRC^{m}}$$

(1B) $$\frac{\text{dC}}{\text{dt}}=\frac{eaRC^{m+1}}{1+ahRC^{m}}\;\text{- dC}$$

In this model, *R* is the resource (prey) and *C* is the consumer (predator). The parameters are intrinsic growth rate of the prey (*r*), prey carrying capacity (*K*), space clearance rate (functional response parameter, *a*), handling time (*h*), mutual interference (*m*), predator conversion efficiency (*e*), and predator natural mortality rate (*d*). The predator zero net growth isocline for this model is $C={\left(\frac{\;\text{-}\;d}{aR(\text{dh - e)}}\right)}^{\text{1}\;/\text{m}}$ The prey isocline requires a numerical solution. We use this model to set the stage for the evolution of prey imperiled by an invasive predator. We do not consider the evolution of predators in this study because invasive predators are likely to start out few in number and thus show rates of evolution that are slower than their prey.

We linked prey body size to prey intrinsic growth rate and the functional response parameters that drive prey deaths (space clearance rate and handling time) using allometric functions. Currently, one of the more complete empirical descriptions of the relationships between body size and consumer–resource model parameters is for protists. We therefore used a dataset on a wide selection of protists consuming algae and other heterotrophic protists in laboratory settings to estimate model parameters and trait‐parameter links (DeLong et al., 2015) (Table 1). The allometric functions for protists are qualitatively in line with expectations for most taxa, so we view the protist scalings as a generic set of body size–parameter relationships. Nonetheless, the degree to which these scalings would mirror the scalings of other taxonomic groups is unknown.

**Table 1:** Linear models estimating the joint effects of predator and prey body size (µm^3^ cell volume) on functional response parameters, based on data from (DeLong et al., 2015)

	Estimate	*SE*	*t*	*p*
log(a) ~ intercept +α*log(Predator volume) + β*log(Prey volume)				
Intercept	−13.23	0.84	−15.76	<0.001
Predator volume exponent (*α*)	0.82	0.097	8.47	<0.001
Prey volume exponent (*β*)	0.20	0.11	1.79	0.078
log(h) ~ intercept +α *log(Predator volume) + *β* * log(Prey volume)				
Intercept	−2.30	0.73	−3.18	0.002
Predator volume exponent (*α*)	−0.69	0.083	−8.25	<0.001
Prey volume exponent (*β*)	0.72	0.096	7.53	<0.001

We also linked prey body size to predator conversion efficiency because prey size determines the amount of energy a predator acquires per prey captured, influencing predator birth rate. Specifically, the conversion efficiency can be written as *e* = GGE * M_R_/*M*_C_, where GGE is the predator's gross growth efficiency, *M*_R_ is prey size, and *M*_C_ is predator size. We use GGE = 0.4, which is typical of some protist predators (Rogerson, 1981). Thus, prey body size indirectly feeds back to the prey population by altering the growth rate of the predator population. We included a relatively low level of mutual interference (*m* = −0.05) to add some stabilization to the dynamics because many consumers show some level of interference (Arditi, Callois, Tyutyunov, & Jost, 2004; DeLong & Vasseur, 2011).

We chose an intermediate‐sized predator (1×10^5^ μm^3^ cell volume) foraging on prey (1×10^4^ μm^3^ cell volume) that would be typical for this size of predator (i.e., prey 1/10th the size of its predator). Predator and prey body size are typically correlated across predator–prey pairs (Brose et al., 2006; Gibert & DeLong, 2014), and both predator and prey sizes are expected to influence functional response parameters (DeLong & Vasseur, 2012a, 2012b; Rall et al., 2012). We therefore assessed the effect of prey body size on the functional response parameters with linear models where the logarithms of predator and prey body size are predictor variables and the logarithms of space clearance rate and handling time are the response variables (Table 1). We then used the exponents of the prey body size effect in the allometric functions to drive a change in functional response parameters as the prey size changes, with the predator size held constant. Prey body size also is negatively related to growth rate independent of predator size. We set the growth rate at the starting body size at 3 da^‐1^ and set up an allometric function to change growth rate with prey size following previously observed scalings: $r=8.83M_{\text{R}}^{\;\text{-}\;0.2}$ (DeLong et al., 2015). Similarly, we determined the predator's death rate using a previously identified allometric scaling relationship for protist mortality rates: $d=5.62M_{\text{R}}^{-0.29}$ (DeLong et al., 2015). Given this prey size‐dependent parameterization, we then calculated the predator and prey zero net growth isoclines for prey sizes of 1 × 10^3^, 1 × 10^4^, and 1 × 10^5^ µm^3^ cell volume.

### 2.2 Gillespie eco‐evolutionary models (GEMS)

Gillespie eco‐evolutionary models are an evolutionary version of the standard Gillespie algorithm that simulates ordinary differential equation (ODE) models by turning model rates (represented by model terms) into stochastic, discrete events (DeLong & Gibert, 2016; DeLong & Luhring, 2018; Gillespie, 1977; Yaari, Ben‐Zion, Shnerb, & Vasseur, 2012). GEMs add evolution by allowing event probabilities to depend on phenotypic traits, generating selection on the model population that is a direct computational analog to natural selection. That is, the trait of individuals drawn during each iteration of the simulation (each iteration involves one randomly chosen individual, one event, and the passage of a random amount of time) influences which event occurs (e.g., prey birth, predation) and thus the individual's fitness. The result is a gradual evolution of the population toward trait distributions that increase survival and reproduction. See Figure 1 in DeLong and Gibert (2016) for a schematic overview of how GEMs work. As an approach to modeling eco‐evolutionary dynamics, GEMs have a few distinct advantages over other modeling approaches: (a) Making direct links between traits and the components of fitness (births and deaths) obviates the need for writing out explicit fitness gradients, which get more challenging when multiple traits or trait‐parameter links are involved, (b) tracking trait distributions allows incorporation of current levels of heritable trait variation at all times, such that the effect of selection on trait variation influences further evolution, (c) allowing for the effects of demographic and individual stochasticity and genetic drift on trait evolution, and (d) being simpler and more computationally efficient than individual‐based models by tracking distributions instead of the fate of individuals.

**Figure 1:**
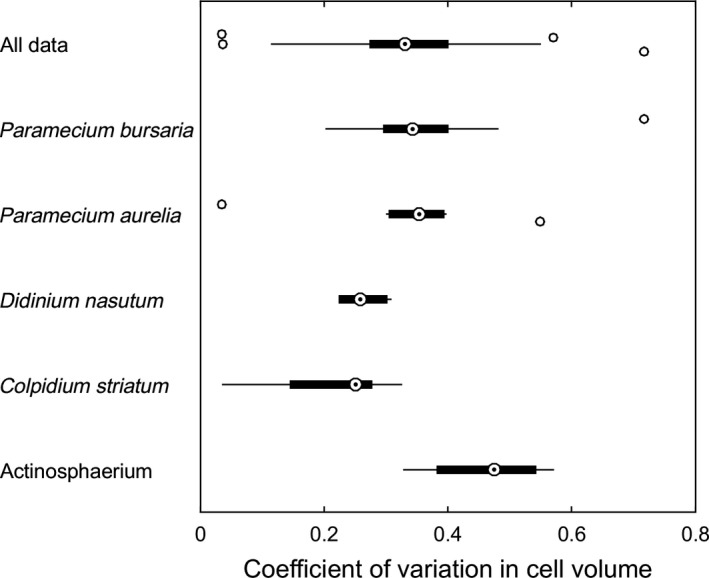
Variation in cell volume for protists. Each point in the distributions is a separate sample for the indicated species, taken from different studies, different treatments, or different days within a study. On the whole, the amount of cell volume variation in a population is highly variable, but the approximate center of the observations is CV = 0.3. We therefore use this value in our initial GEM analysis. Sources are *Paramecium bursaria* (Luhring & DeLong, 2017), *Paramecium aurelia *(DeLong, Hanley, & Vasseur, 2014; DeLong & Vasseur, 2012b), *Didinium nasutum* (DeLong et al., 2014), *Colpidium striatum* (DeLong & Vasseur, 2012b; Jiang & Morin, 2005), and *Actinosphaerium* sp. (DeLong, 2012)

We assigned traits to newly formed offspring in the GEMs by randomly drawing the trait from a distribution of potential traits that depend on the parent trait, the heritability of that trait, and the level of trait variation in the population. The distribution from which the trait is drawn has a mean of the expected value of the offspring trait that is determined by a parent–offspring regression, following the approach of DeLong and Luhring (2018). In short, the expected value of a particular offspring's trait (*o*_c_) is related to the parent trait through the equation of a parent–offspring regression: $E\left[o_{c}\right]=h^{2}p_{c}+\bar{p}\left(1\;\text{-}\;h^{2}\right)$,where *h* ^2^ is narrow‐sense heritability, $\bar{p}$ is the mean of the parent population, and *p*_c_ is the current parent trait. We also assign a level of variance around the expected value of the offspring. The unexplained noise (*ν*) around the expected value of the offspring trait has a standard deviation of $\nu_{\sigma o}=\sigma_{p}\sqrt{1-{\left(h^{2}\right)}^{2}}$,where *σ_p_* is the standard deviation of the trait in the parent population. We use the heritability‐weighted mean of the initial and current population variance to estimate the current *σ_p_*, because use of the current standing variation causes rapid, stochastic loss of genetic variance through time. We then randomly draw offspring traits from a lognormal distribution with mean *E*[*o*_c_] and standard deviation *ν_σo_*. This approach is different from previous quantitative genetic approaches that model changes in means or whole distributions of traits through time rather than identifying the value of a single individual trait that is added to a population (Chevin, 2015; Coulson, Plard, Schindler, Ozgul, & Gaillard, 2015).

Because populations may go extinct by chance when their abundances approach zero, we defined evolutionary rescue as a decrease in the proportion of populations (simulation runs) that went extinct through time. We visualized this as cumulative extinction curves that integrate both the temporal aspect of extinctions and the proportion of populations still extant at any point in time. In our simulations, rescue increases the chance of persistence (Gomulkiewicz & Shaw, 2013; Mellard, Mazancourt, & Loreau, 2015; Northfield & Ives, 2013; Schiffers, Bourne, Lavergne, Thuiller, & Travis, 2013) rather than generating a condition where the rescued population returns to a prestress state after an exponential decline (e.g., a U‐shaped rescue curve; Gomulkiewicz & Holt, 1995; Orr & Unckless, 2014). We illustrate this stochastic extinction process and our cumulative extinction curves by running an initial set of simulations with the allometrically determined parameters but with the predator's death rate set at multiples (i.e., 0.5, 0.75, 1, and 1.5 times) of the allometrically predicted value. We also set trait variation to zero in these simulations so that they are nonevolutionary and show only the effects of stochasticity on the dynamics and likelihood of extinction.

We ran the GEM models in four sets to assess the effects of (a) the specific trait‐parameter links used and the effect of ecological pleiotropy, (b) the indirect effects of prey evolution on predator population growth, (c) the role of heritable trait variation, and (d) the amplitude of oscillations due to variation in prey productivity. In these simulations, prey size was initiated as a distribution with a coefficient of variation (CV) of 0.3 (i.e., typical of body size distributions in protists, see Figure 1; see data in Table S1), unless otherwise specified. We set narrow‐sense heritability (*h* ^2^) at 0.75 and prey carrying capacity at 200, unless otherwise specified. No‐evolution controls were run with CV = 0. Each GEM was run 500 times. MATLAB code that runs the GEM simulations in Figure 4 is available in Appendix 1.

#### 2.2.1 Ecological pleiotropy and indirect effects

In the first set of simulations, we evaluated the potential for evolution of prey body size to alter population dynamics through its link with two separate model parameters: the functional response parameter *a* and prey growth rate (*r*). The allometry of handling time (*h*) was included in our models but for each iteration of the GEM, the handling time was the current population‐level average rather than the handling time predicted for the current prey individual. We did this because time spent handling occurs after predation events, so the handling time experienced by the current predator would be the average of the handling times that could have been consumed previously, which can be approximated by the average of all handling times in the population. We then ran a GEM where body size was connected to *a* and *r* at the same time (an ecologically pleiotropic model). We ran these scenarios again with the conversion efficiency (*e*) changing in response to prey body size changes, generating an indirect effect on the prey through the growth rate of the predator.

#### 2.2.2 Heritable trait variation

We then ran the ecologically pleiotropic GEM for different levels of initial trait variance (i.e., CV = 0, 0.2, 0.4, 0.6, and 0.8) to determine what levels of variation are sufficient for generating evolutionary rescue. Other parameters were set as indicated above.

#### 2.2.3 Prey population oscillations

Finally, resource levels in consumer–resource models such as Equation (1A), (1B) can strongly influence the system's stability (Rosenzweig, 1971), thereby influencing the probability of extinction by determining how close populations come to zero during their cycles. Thus, we vary the carrying capacity of the prey from 50 to 400. This range of *K* values changes our system from one where the prey nearly always persist (and thus do not need rescue) to one where both the predator and the prey nearly always go extinct. The behavior of the system over this range of *K* changes from one with a fixed‐point equilibrium to one with strongly oscillating but dampening dynamics. By doing this, we allow the typical variation in system dynamics to interact with the evolutionary rescue process. We ran the GEM at each level of *K* with and without evolution, with the evolution version again being the fully pleiotropic model. To remove evolution, parameters were fixed at their initial, allometrically predicted, values.

## 3 RESULTS

**Figure 2:**
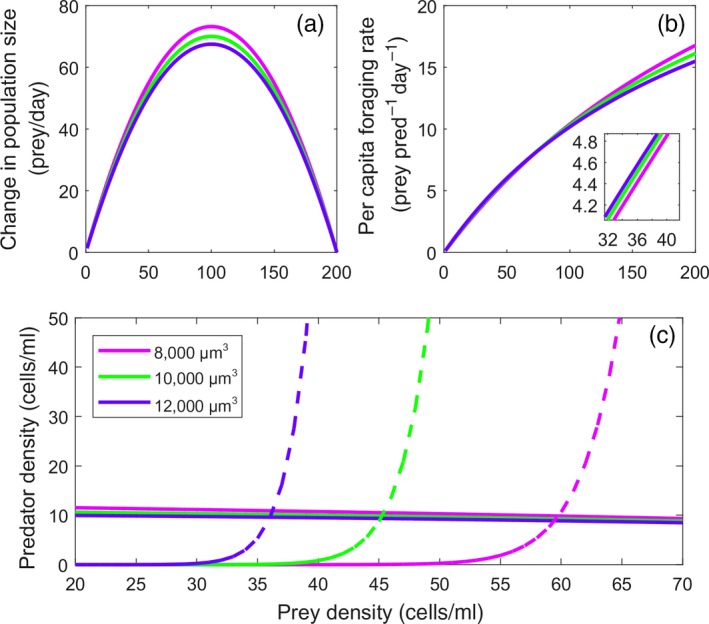
Prey mass affects growth, foraging rates, and equilibrium abundances. (a) Change in population size with population size for small (0.8×10^4^ µm^3^ cell volume, purple), medium (1×10^4^, green), and large (1.2×10^4^, blue) prey. (b) Variation in the functional response for small, medium, and large prey. Although difficult to see, the purple curve has a shallower slope than the other curves at low prey density (inset), indicating that smaller prey may be consumed relatively less when rare. (c) Zero net growth isoclines for predator (dashed) and prey (solid) for Equations (1A), (1B) with three levels of prey size. There is little variation in the prey isocline relative to the predator isocline. The predator isocline moves to the right as prey size declines, indicating that the equilibrium population size for prey is higher for smaller individuals

The relationships between prey mass and model parameters indicate that prey is most likely to evolve smaller body sizes, with the net effect depending on which trait‐parameter links we include in the model (Figure 2a,b). When linked to intrinsic rate of growth (*r*) and the initial slope of the functional response (*a*), prey should evolve toward smaller sizes because they reproduce faster and get consumed at a slower rate, respectively. In addition, the indirect effect of prey size on predator reproduction indicates that smaller prey limits predator population growth and reduces predation rates. When the direct and indirect effects of changes in prey size act together (ecological pleiotropy), the net effect would depend on the magnitude and sign of each trait‐parameter link in the context of the model. Regardless of the net direction of selection on prey size, the predator and prey isoclines indicate that in this system, smaller prey has a higher equilibrium abundance than larger prey, mainly due to the predator isocline shifting to the right (Figure 2c). The predators also have higher equilibrium abundance with smaller prey, although this effect is very small relative to the prey abundance effect. We would thus expect that when stochastic extinctions are possible, evolution toward smaller size would generate an increased probability of persistence.

**Figure 3:**
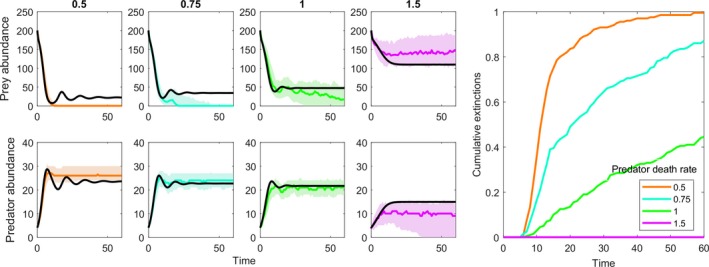
How proximity of an equilibrium influences the probability of stochastic extinctions. Prey abundance (top row) and predator abundance (bottom row) from a no‐evolution GEM with allometric parameterization (see text). The columns show the parameterization with the predator's death rate being 0.5, 0.75, 1, and 1.5 times that of the allometrically predicted rate. The far right panel shows the cumulative probability of extinctions as time passes in these simulations. The solid black lines show the standard ODE solution of Equation (1A), (1B), and the solid color lines show the median solution (shaded areas show the middle 50%) using a no‐evolution GEM that allows stochastic extinctions to occur. The key observation is that increasing the prey equilibrium reduces the chance of stochastic extinctions. Thus, evolutionary rescue may operate by raising equilibrium densities and reducing the chance of going extinct

Prey populations showed a strong tendency to go extinct when their abundances veered closer to zero (Figure 3). The prey populations oscillated around higher levels with increasing predator mortality. This upward shift decreased stochastic extinctions, such that at 0.5 of the allometrically predicted death rate, 100% of the runs went extinct in the 60 time steps, and at 1.5 times the predicted death rate, extinctions almost never occurred. This pattern shows that systems that might be predicted to persist given that they have positive equilibrium abundances still can suffer stochastic extinctions, and thus, evolution that moves an equilibrium higher and reduces stochastic extinctions is a form of evolutionary rescue.

**Figure 4:**
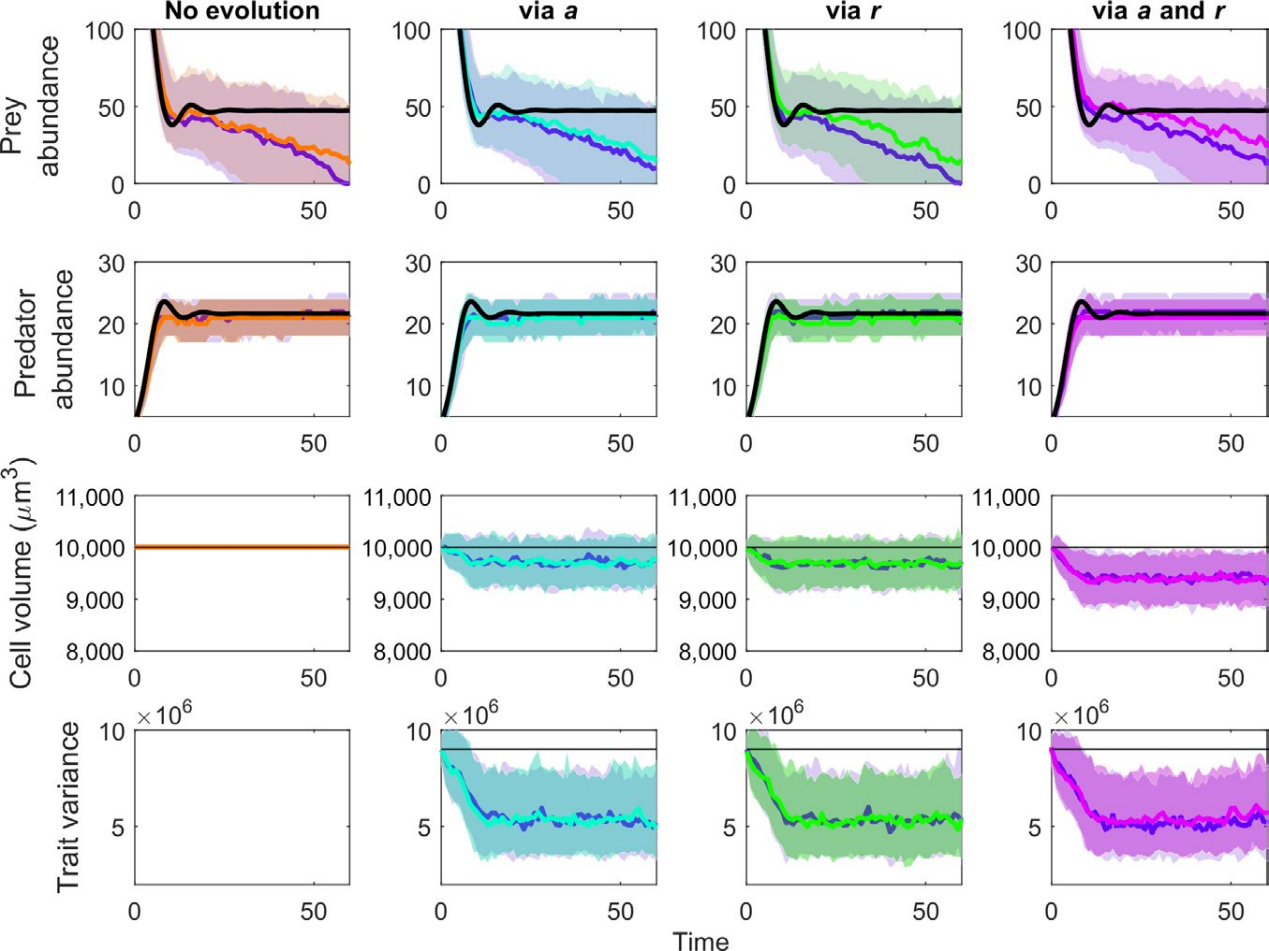
Eco‐evolutionary dynamics of predator, prey, and prey body size where the body size is connected to different functional aspects of the predator–prey interaction. Each column shows dynamics for different scenarios. In column 1, there is no trait variation, so no‐evolution is possible. In columns 2‐4, prey size is connected to the functional response parameter space clearance rate (*a*), prey growth rate (*r*), or both, respectively. In the top two rows, the solid black line indicates the no‐evolution standard ODE solution of Equations (1A), (1B). In each box, two scenarios are shown. The bold blue line and light blue shading show the median and middle 50% of simulations for the GEMs without the indirect effect of predator conversion efficiency (*e*) on the dynamics. The other bold color lines and shaded areas show the median and middle 50% of simulations of the GEMs with the indirect effect of predator conversion efficiency. Each column is given a different color to link the dynamics in this figure with the evolutionary rescue outcomes in Figure 5. From top to bottom, the rows show prey density, predator density, mean trait (cell volume), and variance in the trait

**Figure 5:**
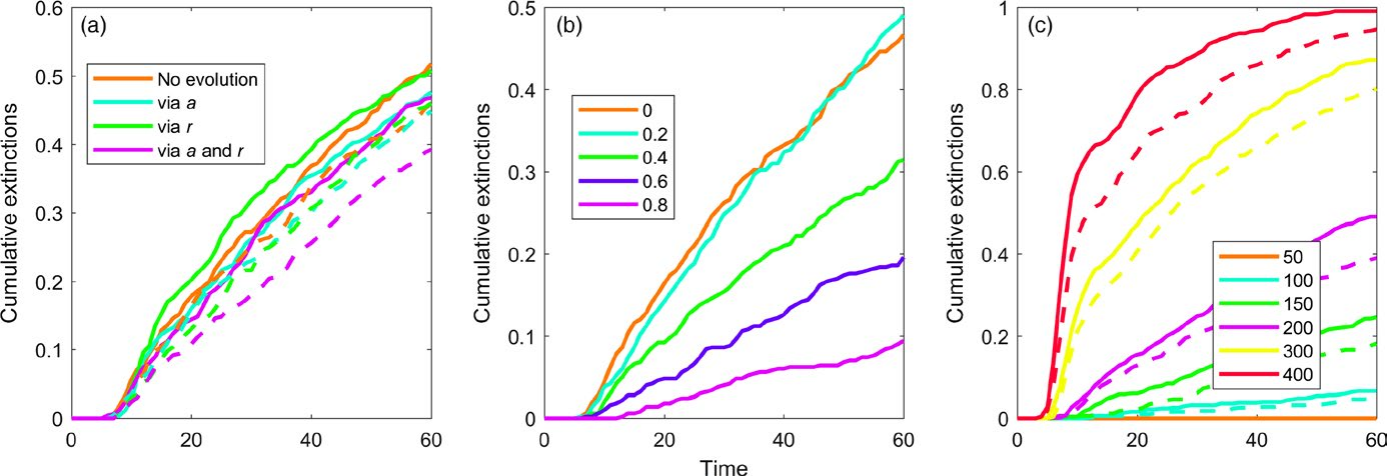
Cumulative extinctions of the prey population across different eco‐evolutionary scenarios. (a) Cumulative extinctions across versions of our model, including a no‐evolution scenario (trait variance set at 0), evolution through the space clearance rate (a), prey growth rate (*r*), and both traits combined. Solid lines are for models without and dashed lines are for models with the indirect effect of prey size on predator conversion efficiency. (b) Cumulative extinctions for the fully ecologically pleiotropic model for increasing levels of heritable trait variation (0–0.9). (c) Cumulative extinctions for the fully ecologically pleiotropic model for increasing levels of carrying capacity (K)

**Figure 6:**
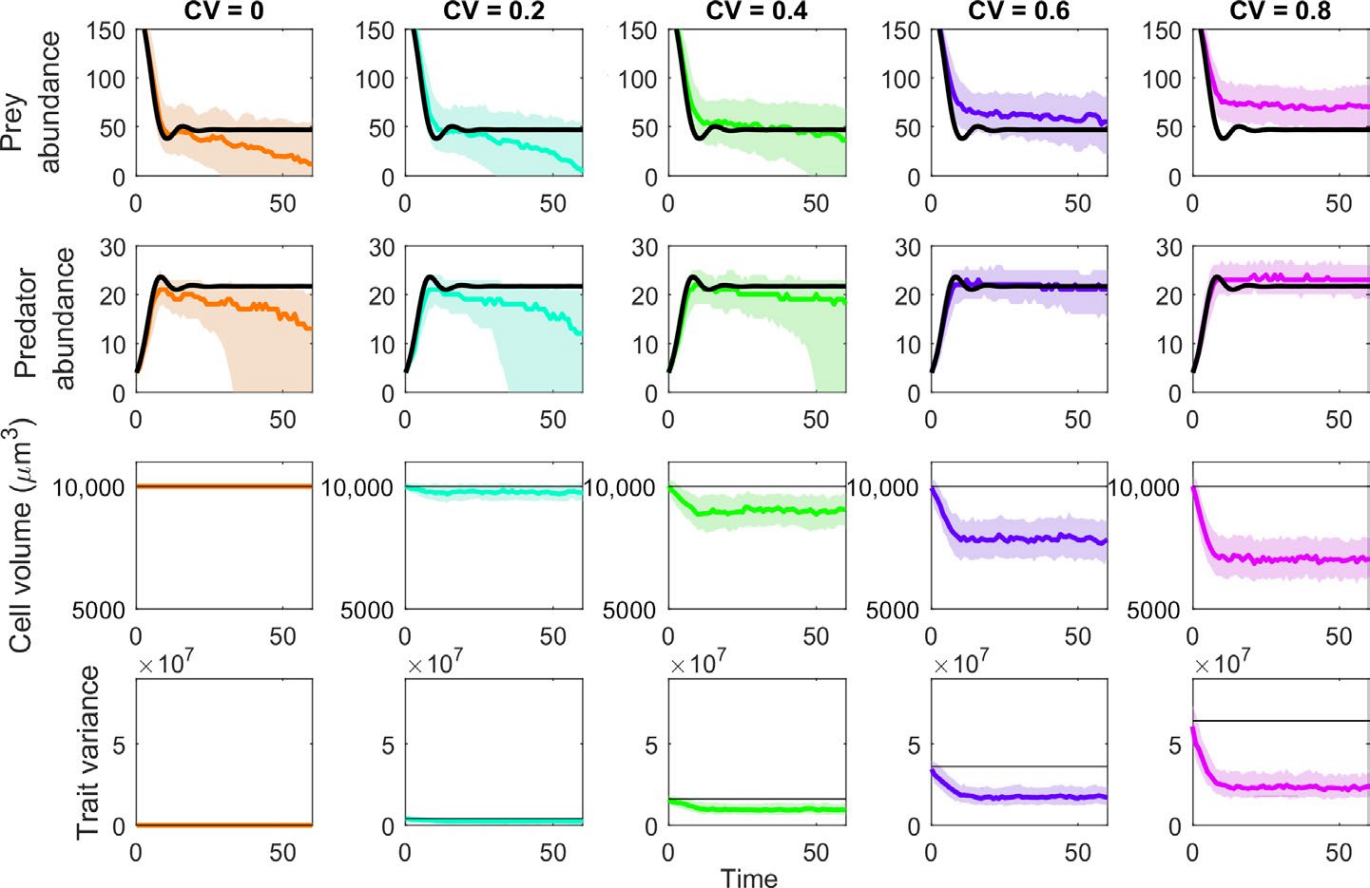
Eco‐evolutionary dynamics of predator, prey, and prey body size where the amount of initial heritable trait variation (CV) increases from zero (no‐evolution) to 0.8. Higher amounts of variation promote a greater degree of evolution and fewer stochastic extinctions. At CV = 0.6 or above, both the predator and the prey populations are rescued by evolution. Figure set up the same as in Figure 4

In our evolutionary simulations, prey populations evolved smaller sizes (Figures 4 and 6 show eco‐evolutionary dynamics; Figure 5 shows summary of outcomes). Evolution toward smaller body size is consistent with expectations because smaller size reduces predation (given the relationships in Table 1) and increases prey growth rate. Nonetheless, even with this evolution, there was little indication that the observed size change would lead to evolutionary rescue of the prey when size was connected to only one parameter (Figure 5a). In the ecologically pleiotropic model, with prey size linked to space clearance rate and prey growth rate, the magnitude of prey size evolution increased, but there was still little increase in the probability of persistence (Figure 5, column 5, Figure 5a). When the indirect effect of prey body size change on the predator's conversion efficiency was included, however, evolution did lead to a noticeable increase in the probability of persistence through time (Figure 4).

Using the pleiotropic GEM model for which prey size influences space clearance rate, prey growth rate, and the indirect effects through predator conversion efficiency, increasing initial trait variance increased the probability of rescue (Figures 5b and 6). The results suggest that CVs in the range of 0.3–0.4 are sufficient to enable trait evolution and some degree of increased prey persistence in our system, but rescue becomes nearly certain when CVs reach ~0.8. At these high levels of trait variation, rescue also boosts prey population sizes high enough to foster an increased abundance of the predators.

**Figure 7:**
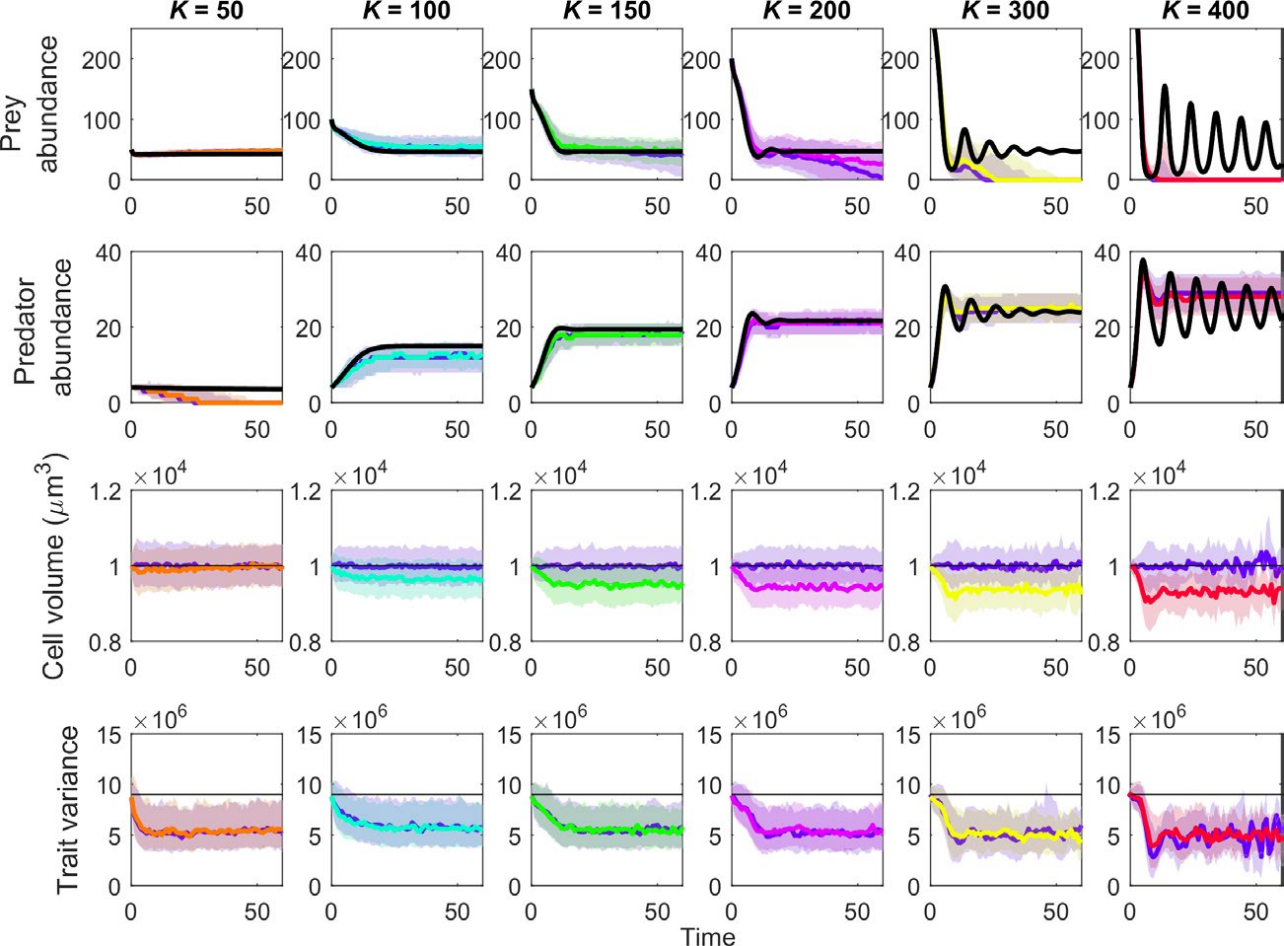
Eco‐evolutionary dynamics of predator, prey, and prey body size where prey carrying capacity ranges from 50 to 400. Figure set up the same as in Figure 4

Again using the fully pleiotropic GEM, variation in system stability strongly influenced both the need for evolutionary rescue and the possibility of its occurrence (Figures 5c and 7). As carrying capacity increased, the system changed from a stable one that rapidly moved to a fixed‐point equilibrium to one with high amplitude but dampening oscillations. Along this gradient, there was an increase in the amount of trait evolution, with prey body sizes decreasing to a greater extent in the more unstable systems. When carrying capacities were low, system oscillations were dampened, leading to decreased likelihood of prey extinctions. When carrying capacities were high, strong oscillations generated high probabilities of extinctions. Only at intermediate levels of carrying capacity were there both a need and an opportunity for evolutionary rescue.

## 4 DISCUSSION

As species continue to invade new regions around the world, more and more prey species will encounter novel predators that have the potential to greatly reduce their numbers (Albins & Hixon, 2008; Buba et al., 2017; Dorcas et al., 2012; Wiles, Bart, Beck, & Aguon, 2003). In some of these cases, naïve prey populations may become imperiled and go extinct (Savidge, 1987). Yet predation is a powerful selective agent with the potential to select for more resistant forms that could persist despite the increased predation pressure. As such, evolutionary rescue could safeguard some invaded communities from species loss (Faillace & Morin, 2016; Gillis & Walsh, 2017; Vander Wal et al., 2013).

Evolutionary rescue of prey imperiled by invasive predators depends on the existence of heritable trait variation that can be functionally linked to important aspects of the predator–prey interaction (trait‐parameter links). For example, prey traits that reduce predator searching effectiveness will limit prey mortality and foster prey persistence (e.g., Kiesecker & Blaustein, 1997; Imura et al., 2003). Similarly, traits that increase prey growth rate could allow prey to replenish their populations more quickly (e.g., Gillis & Walsh, 2017). Our results suggest that one important aspect of evolutionary rescue might be the existence of positive ecological pleiotropy, where traits influence the multiple functions that combine to increase the rate of evolution (DeLong, 2017; DeLong & Gibert, 2016). The pleiotropic effects in our model had a net positive effect on the pace of evolution, and by interacting to increase the prey equilibrium (Figure 2), led to an increase in the probability of persistence (Figure 5a). However, ecological pleiotropic effects also could be antagonistic, and if so, such effects could make evolutionary rescue much less likely. It is not clear whether we should generally expect positive or antagonistic ecological pleiotropy, and it is therefore difficult to make predictions about the role of ecological pleiotropy in evolutionary rescue more generally.

Our results also suggest an important role for indirect effects of prey traits on the predator's demography. In our model, smaller prey yield less energy per individual to the predators. Therefore, when evolution selects for smaller size, the conversion efficiency of the predators declines, reducing their reproductive rate. This effect is indirect because the positive effect on the prey population is generated via the future abundance of the predators. This result implies that rescue might depend on how the evolution of traits feeds back into the environmental or food web context in which the imperiled prey resides.

Since evolution cannot proceed without heritable variation, limits on the amount of variation will preclude evolutionary rescue in the absence of future mutations (Bell, 2013; Lande, 1976). It remains an important open question just how much heritable trait variation there is in nature for traits that could functionally alter ecological dynamics. Our survey of protist cell volumes (Figure 1) suggests that the amount of variation necessary to support an increased chance of persistence (coefficients of variation greater than ~0.3) is readily available in the systems we are using here as a case study. More initial trait variance can increase the probability of rescue greatly, but these values of variation might be exceptionally large (Lande, 1977), so whether other taxa have sufficient underlying trait variation needs further scrutiny. Moreover, strong rescue effects supported by high initial trait variation led to increased population abundance of the predator, buffering it against extinction (Yamamichi & Miner, 2015), which could increase the effects of invasive predators on other imperiled prey.

The underlying need—from a conservationist's perspective—for evolutionary rescue depends, in the first place, on some reasonable likelihood of population extinction. By varying the carrying capacity of our system, we harnessed the paradox of enrichment (Rosenzweig, 1971) to generate variation in the amplitude and duration of oscillations and the background probability of stochastic extinctions (Figure 7). Because natural systems vary greatly in productivity, it is possible that predator–prey interactions involving exotic predators and naïve prey will vary substantially in dynamic stability and the likelihood of prey extinction, and thus the need for rescue. Some interactions may never put prey populations in jeopardy and will be accompanied by mild prey evolution, while others will generate strong oscillations that will tend to drive both predator and prey extinct despite substantial evolution of prey traits (Figure 7). It is in the area of intermediate stability where evolutionary rescue is likely to have its greatest impact stabilizing the populations of imperiled prey (Figure 7).

Some caveats of our analysis are that we have used one possible model with a range of trait‐parameter links and environmental productivity levels, which we parameterized for a subset of taxa and traits. This is in large part due to limitations on our empirical understanding of how potentially evolving traits are empirically linked to predator–prey interactions. Further exploration of other systems, including systems with more types of predator and prey, depends on developing detailed empirical descriptions of how traits are linked to multiple aspects of predator–prey interactions (trait‐parameter links). Given that exotic predators include a huge range of taxa including fish, insects, mammals, reptiles, amphibians, crustaceans, and molluscs, we need considerable improvement in our understanding of the functional consequences of evolving traits. Nonetheless, we think that the empirical links between prey size and model parameters in protists are likely to be qualitatively similar to other groups (DeLong et al., 2015; Rall et al., 2012; Weitz & Levin, 2006), suggesting that our results may serve as a good starting point for future assessments of the potential for evolutionary rescue and the roles of ecological pleiotropy and indirect effects. We also note that although body size can account for substantial amounts of variation in predator–prey interactions, it clearly cannot account for all of it (Kalinoski & DeLong, 2016; Rall, Kalinkat, Ott, Vucic‐Pestic, & Brose, 2011). Thus, other types of traits, such as defensive structures, predator avoidance behaviors, or life‐history strategies, need consideration as candidate traits that could evolve and lead to rescue.

We evaluated the roles of ecological pleiotropy, indirect effects, heritable trait variation, and population oscillations in determining the potential for evolutionary rescue using Gillespie eco‐evolutionary models (GEMs). GEMs are a computational analog to natural selection wherein traits determine the likelihood of fitness‐related events such as births and deaths. Because of the stochastic nature of GEMs, processes such as demographic stochasticity, individual demographic stochasticity, and genetic drift—all of which should be important constraints on evolutionary rescue in small populations—can play out. These models thus provide increased realism relative to classic deterministic and individual‐based quantitative genetic models, which often have been used to study evolutionary rescue (Bell & Gonzalez, 2009; Carlson et al., 2014; Gomulkiewicz & Holt, 1995; Gomulkiewicz & Shaw, 2013; Gonzalez et al., 2013; Jones, 2008; Kopp & Matuszewski, 2014; Lindsey et al., 2013; Schiffers et al., 2013). Our results indicate that evolutionary rescue of imperiled prey through the evolution of prey body size is possible given empirically determined links between prey body size and predator–prey interactions. However, evolutionary rescue is much less likely when only single functional consequences of evolving traits are considered, indirect negative effects on predators do not occur, and when systems show strong oscillations that periodically bring populations to low levels.

For the task of conserving prey at risk of extinction due to introduced predators, it is important to recognize that any number of traits could evolve if they are functionally linked to the processes leading to low abundance and extinction. If those traits show ecologically pleiotropic effects and/or indirect effects on the predators, however, the pace of evolution could increase or decrease. Thus, our expectation that rescue might actually influence any particular conservation scenario should be tempered by the realization that we generally do not know all the ways in which a particular trait influences species interactions and life histories. Furthermore, the traits that do evolve are more likely to be those that show relatively high levels of variation. Although trait‐parameter links and standing variation are generally out of the control of managers, it may be possible to augment trait variation in general by manual outcrossing or transplanting individuals across populations or subpopulations. Doing so could increase the chance that some trait with the requisite pleiotropic effects on the predator–prey interaction could undergo selection and contribute to evolutionary rescue. However, our results also show that evolutionary rescue may have the undesired outcome of bolstering the population of the invasive predator. Thus, managers should be prepared for the possibility that any successful case of evolutionary rescue may exacerbate circumstances for other species at risk from invasive predators.

## CONFLICT OF INTEREST

None declared.

## DATA ACCESSABILITY

The cell volume data used in this study are available as a supplementary file. The protist allometry data are freely available online.
